# Hantavirus Pulmonary Syndrome in a COVID-19 Patient, Argentina, 2020

**DOI:** 10.3201/eid2804.211837

**Published:** 2022-04

**Authors:** Rocío M. Coelho, Natalia Periolo, Carolina Perez Duhalde, Daniel O. Alonso, Carla M. Bellomo, Marisa Corazza, Ayelén A. Iglesias, Valeria P. Martinez

**Affiliations:** Administración Nacional de Laboratorios e Institutos de Salud Dr. Carlos G. Malbrán, Buenos Aires, Argentina (R.M. Coelho, N. Periolo, D.O. Alonso, C.M. Bellomo, A.A. Iglesias, V.P. Martinez);; Hospital Interzonal General de Agudos, Buenos Aires (C. Perez Duhalde);; Instituto Biológico “Tomás Perón,” Buenos Aires (M. Corazza)

**Keywords:** COVID-19, coronavirus disease, SARS-CoV-2, severe acute respiratory syndrome coronavirus 2, viruses, respiratory infections, zoonoses, vaccine-preventable diseases, hantavirus, Argentina

## Abstract

We describe a patient in Argentina with severe acute respiratory syndrome coronavirus 2 infection and hantavirus pulmonary syndrome (HPS). Although both coronavirus disease and HPS can be fatal when not diagnosed and treated promptly, HPS is much more lethal. This case report may contribute to improved detection of co-infections in HPS-endemic regions.

The current coronavirus disease (COVID‐19) pandemic, caused by severe acute respiratory syndrome coronavirus 2 (SARS‐CoV‐2), has resulted in substantial illness and death rates worldwide. Orthohantaviruses are zoonotic viruses responsible for another severe respiratory infectious disease in the Americas, hantavirus pulmonary syndrome (HPS). Although humans generally become infected with HPS through inhaling excreta generated by infected rodents, person-to-person transmission has been well documented in Argentina and Chile (*1*–*3*). Humans become infected with SARS‐CoV‐2 and orthohantaviruses in similar ways, through inhaling contaminated aerosols, and can have onset of similar respiratory syndromes. Despite these similarities, the incubation period is shorter in COVID-19 patients (2–14 days) than in HPS patients (7–45 days). Furthermore, at the time the case we describe was reported, the cumulative case-fatality rate for COVID-19 in Argentina was 2.7% (*4*); for HPS, it was 22%–40% (*5*).

HPS is characterized by the onset of symptoms such as fever, myalgia, cough, dyspnea, diarrhea, and sweating. Rapid progression to shock or respiratory distress can occur within hours. Symptom-based therapy with oxygen and ventilatory or circulatory support is needed (*6*,*7*).

We describe a case of SARS‐CoV‐2 and Andes virus co-infection in central Argentina. The patient, a 22-year-old woman without relevant pathologic records, sought care at a local hospital in November 2020 for fever, headache, myalgia, and gastrointestinal manifestations. A nasopharyngeal swab sample tested positive for SARS-CoV-2 by reverse transcription PCR at the Instituto Biológico “Tomás Perón” (Appendix). Five days after the onset of fever, the patient’s clinical status worsened, and she was admitted to the hospital. Clinical laboratory findings at admission indicated thrombocytopenia, high leukocyte count, lymphopenia, and elevated hepatic enzymes (Appendix). Computed tomography revealed bilateral pleural effusion associated with interstitial infiltration, and capillary filtration with slight peripheral pulmonary ground-glass opacity (Figure; Video).

**Figure:**
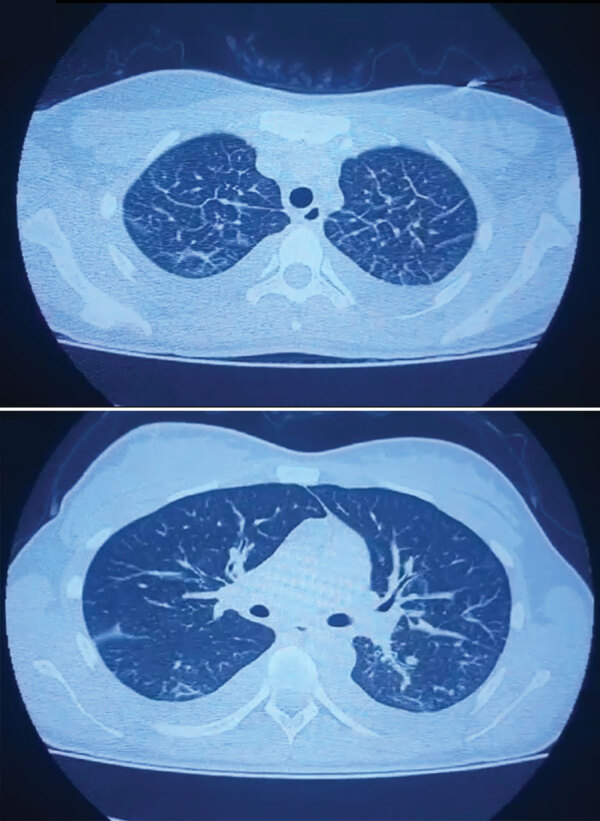
Computed tomography scan results on the second day of hospitalization (day 7 after fever onset) for a patient with severe acute respiratory syndrome coronavirus 2 and hantavirus co-infection, Argentina, 2020, showing pleural effusion, interstitial compromise, vascular congestion, and glass-ground opacities.

**Video:** Live-capture computed tomography scan results on the second day of hospitalization (day 7 after fever onset) for a patient with severe acute respiratory syndrome coronavirus 2 and hantavirus co-infection, Argentina, 2020.

Within a few hours after admission, the patient had onset of marked respiratory distress. She was then referred to the intensive care unit for orotracheal intubation and treated with ampicillin/sulbactam and azithromycin. The epidemiologic investigation established that the patient resided in a hantavirus-endemic area. Consequently, HPS was suspected, despite the COVID-19–positive diagnosis. According to the confirmation criteria used by the Hantavirus National Reference Laboratory (*8*), Andes virus infection was confirmed by the detection of specific IgM and IgG by ELISA and genomic viral RNA by quantitative reverse transcription PCR in blood (Appendix). 

Three days after the co-infection was confirmed, the patient was extubated and progressed favorably. Twenty days after onset of symptoms, she was discharged from the hospital.

To determine the viral genotype of Andes virus, we conducted a nucleotide sequence analysis from 2 partial fragments of viral small (496-bp) and medium (611-bp) segments, and we submitted the sequences obtained to GenBank (accession nos. OL840325 and OL840326). The highest nucleotide identities matched previous published sequences corresponding to Plata genotype of Andes virus (GenBank accession nos. EU564720 [96% identity] and AY101185 [97.8 identity]). This viral genotype is one of the prevalent pathogenic orthohantaviruses circulating in central Argentina and Uruguay (*9*).

Because the incubation period for HPS is longer than that for COVID-19, we might speculate that hantavirus infection occurred before coronavirus infection. The respiratory distress syndrome appeared 5 days after the onset of fever, which coincided with the characteristic prodromal period described for HPS. This condition, during the incubation period of HPS, could have induced a higher susceptibility to COVID-19. Because HPS can evolve rapidly to respiratory failure in most patients with severe disease, resulting in high case-fatality rates, alerting health-care workers from HPS-endemic areas is warranted to detect co-infections in the context of the COVID-19 pandemic. In particular, at least 2 genotypes of Andes virus can be transmitted person-to-person, and these species are prevalent in 2 of the 3 hantavirus-endemic regions of Argentina (*10*).

In conclusion, we detected co-infection with SARS-CoV-2 and Andes virus causing HPS in a patient from a hantavirus-endemic area. Clinicians should be aware of the possibility of co-infection for patients originating, residing, or traveling in hantavirus-endemic areas.

Appendix Additional information about hantavirus pulmonary syndrome in a COVID-19 patient, Argentina, 2020.
