# Supplementary figures and images for: Hantavirus Pulmonary Syndrome in a COVID-19 Patient, Argentina, 2020
Source: Emerg Infect Dis. 2022 Apr;28(4):876–8. doi: 10.3201/eid2804.211837 (PMC8962894; doi:10.3201/eid2804.211837)

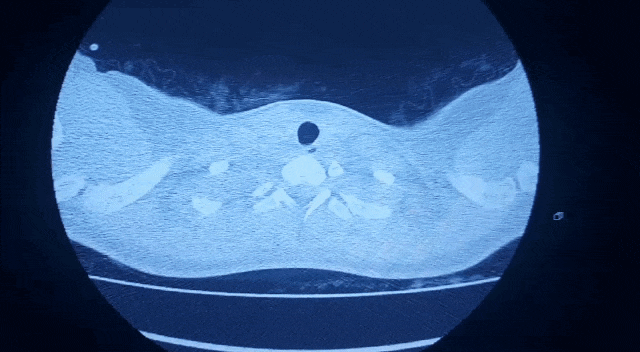

Supplement: Supplementary file 1 [file 21-1837-V.gif]
