# Supplementary material for: Hantavirus Pulmonary Syndrome in a COVID-19 Patient, Argentina, 2020
Source: Emerg Infect Dis. 2022 Apr;28(4):876–8. doi: 10.3201/eid2804.211837 (PMC8962894; doi:10.3201/eid2804.211837)
Supplement: Appendix — Additional information about hantavirus pulmonary syndrome in a COVID-19 patient, Argentina, 2020. [file 21-1837-Techapp-s1.pdf]

Article DOI: https://doi.org/10.3201/eid2804.211837

Hantavirus Pulmonary Syndrome in a COVID-19 Patient, Argentina, 2020

Appendix

Appendix Table. Clinical and virologic laboratory findings*

		Days after onset of symptoms										
Characteristic	Reference value	3	5	6	7	9	10	12	13	14	16	18
Platelet count, cell/mm^3	154–383	ND	55	50	73	100	136	ND	200	ND	332	ND
Hemoglobin, g/dL	11.8–15.5	ND	18.3	16.9	13	8.5	9.6	ND	8.2	ND	8.6	ND
Total leukocytes, 10^{3}µL	4.1–9.8	ND	17.2	12.8	12.3	ND	10.8	ND	12.3	ND	ND	ND
Monocytes, %	5.1–10.1	ND	ND	2	ND	ND	14.3	ND	14	ND	ND	ND
Lymphocyte, %	19.4–44.1	ND	ND	9	ND	ND	23.3	ND	24	ND	ND	ND
Neutrophils, %	41–73.1	ND	ND	58	ND	ND	56.1	ND	60	ND	ND	ND
NLR, %	1–3†	ND	ND	6.4	ND	ND	2.4	ND	2.5	ND	ND	ND
AST, UI/L	0–32	ND	ND	107	70	95	90	68	ND	46	ND	ND
ALT, UI/L	0–31	ND	ND	38	28	56	63	79	ND	58	ND	ND
SARS CoV-2												
RT-PCR gene ORF 1 AB	C_t >38	34.07	ND	ND	ND	ND	35.02	ND	ND	ND	ND	Negative
RT-PCR gene N	C_t >38	29.92	ND	ND	ND	ND	32.05	ND	ND	ND	ND	Negative
Hantavirus												
ELISA IgM	Cut off 0.3	ND	ND	ND	ND	ND	1.64	ND	ND	ND	ND	2.80
ELISA IgG	Cut off 0.55	ND	ND	ND	ND	ND	2.8	ND	ND	ND	ND	3.39
RT-PCR	C_t >38	ND	ND	ND	ND	ND	24.4	ND	ND	ND	ND	ND

*ALT alanine aminotransferase; AST, aspartate aminotransferase, C_t, cycle threshold; ND, not done; RT-PCR, reverse transcription PCR, SARS-CoV-2, severe acute respiratory syndrome coronavirus 2.
†PulmCrit.
